# Integrated Lithium-Rich *y*Li_2_MnO_3_∙(1-*y*)LiNi_1/3_Co_1/3_Mn_1/3_O_2_ Layered Cathode Nanomaterials for Lithium-Ion Batteries

**DOI:** 10.3390/ijms26031346

**Published:** 2025-02-05

**Authors:** Ashraf E. Abdel-Ghany, Rasha S. El-Tawil, Ahmed M. Hashem, Alain Mauger, Christian M. Julien

**Affiliations:** 1 Inorganic Chemistry Department, National Research Center, 33 El Bohouth St., (Former El Tahrir St.), Dokki, Giza 12622, Egypt; achraf_28@yahoo.com (A.E.A.-G.); r2samir@yahoo.com (R.S.E.-T.); ahmedh242@yahoo.com (A.M.H.); 2 Institut de Minéralogie, de Physique des Matériaux et Cosmologie (IMPMC), Sorbonne Université, UMR-CNRS 7590, 4 Place Jussieu, 75752 Paris, France; alain.mauger@sorbonne-universite.fr

**Keywords:** Li-rich compounds, layered oxides, cathode materials, Li-ion batteries

## Abstract

Integrated Li- and Mn-rich layered cathodes *y*Li_2_MnO_3_∙(1-*y*)Li*M*O_2_ (*M* = Mn, Co, and Ni) have shown their ability to deliver specific capacities close to 300 mAh g^−1^, but their significant drawbacks are capacity fading and voltage decay during cycling. In this study, new stoichiometric high-voltage Li-rich oxides with *y* = 0.0, 0.3, and 0.5 are synthesized in identical conditions using a sol–gel method. These compositions were analyzed to determine their optimal configuration and to understand their extraordinary behavior. Their nanostructural properties were investigated using XRD and Raman spectroscopy, while the morphology and grain-size distribution of the samples were characterized by BET, SEM and HRTEM analyses. The electrochemical performances of the integrated Li- and Mn-rich compounds were evaluated through galvanostatic cycling and electrochemical impedance spectroscopy. The best cathode material 0.5Li_2_MnO_3_∙0.5LiNi_1/3_Co_1/3_Mn_1/3_O_2_ had a capacity retention of 83.6% after 100 cycles in the potential range 2.0–4.8 V vs. Li^+^/Li.

## 1. Introduction

Energy storage is a critical component of the energy industry’s strategy to address increasing energy demands and transition towards renewable sources. One of the most effective energy storage systems is the rechargeable lithium-ion battery. These batteries have found widespread applications as power sources in portable electronics, electric vehicles, and large-scale grid energy storage systems. Commercially available Li-ion batteries are typically based on cathodes such as LiCoO_2_, Li(Ni,Co,Mn)O_2_ layered oxides, spinel LiMn_2_O_4_, or polyanionic compounds like LiFePO_4_ [1]. Among these materials, layered oxides have emerged as a solution for achieving high energy density [2,3]. 

However, there is growing interest in the Li- and Mn-rich layered cathodes *y*Li_2_MnO_3_∙(1-*y*)Li*M*O_2_ (*M* = Mn, Co, and Ni), due to their ability to deliver specific capacities close to 300 mAh g^−1^ [4,5,6]. These cathode materials are structurally integrated from solid solutions of Li_2_MnO_3_ and the active Li*M*O_2_ phase, utilizing various combinations of Mn, Ni, and Co [7,8,9,10]. The theoretically electrochemically inactive Li_2_MnO_3_ becomes activated at potentials greater than 4.5 V. This activation leads to structural reorganization, delithiation, and the evolution of molecular oxygen, accompanied by the production of some Li_2_O [11,12]. During charging, this process forms MnO_2_, which undergoes reversible reduction at lower potentials, resulting in the formation of layered Li_x_MnO_2_. Upon cycling, this Li_x_MnO_2_ phase converts into a spinel phase as reported in ref. [13]. The electrochemical properties—such as specific capacity, rate capability and cycling stability—of these integrated cathodes depend on their composition, the activation voltage, the cycling voltage range, and electrode kinetics, including the charge transfer resistance at the electrode/electrolyte solution interface (which itself depends on the composition of the electrolyte solution) [14]. 

Various compositions of Li-ion cathodes have been the subject of extensive research. Amalraj et al. [15] investigated several compositions, reporting a maximum capacity of 250 mAh g^−1^ for *y*Li_2_MnO_3_∙(1-*y*)Li*M*O_2_ (*y* = 0.5).Yu et al. [16] observed a discharge specific capacity of 240 mAh g^−1^ at a current density of 20–30 mA g^−1^ for *y*Li_2_MnO_3_∙(1-*y*)LiMn_1/3_Ni_1/3_Co_1/3_O_2_ (*y* = 0.3). Similarly, Martha et al. [17] reported high specific capacities of 260–280 mAh g^−1^ for Li_1.2_Mn_0.525_Ni_0.175_Co_0.1_O_2_ depending on the voltage range. Despite their high specific capacities, one of the significant drawbacks of Li- and Mn-rich cathode materials is capacity fading and voltage decay during cycling. These issues can be mitigated through surface coatings and by carefully selecting the transition metals in the Li*M*O_2_ structure. Incorporating Mn reduces costs, while Ni and Co improve cycling stability, increase capacity, and lower electrode polarization [18]. 

The current study aims to further explore and identify the optimal combination of *y*Li_2_MnO_2_∙(1-*y*)LiNi_1/3_Co_1/3_Mn_1/3_O_2_, *y*Li_2_MnO_3_∙(1-*y*)Li*M*O_2_, also formulated as Li[Li_(1/3-2x/3)_Ni_x_Co_x_Mn_(2/3-x/3)_]O_2_. The significance of these compounds for modern high-energy Li-ion batteries necessitates more comprehensive research. Advanced studies using precise electrochemical measurements, high-resolution microscopy, and optical spectroscopy could provide deeper insights into the limitations of these electrodes and potential solutions. 

In this study, the new stoichiometric high-voltage Li-rich integrated cathode materials *y*Li_2_MnO_3_∙(1-*y*)LiNi_1/3_Co_1/3_Mn_1/3_O_2_ (where *y* = 0.0, 0.3, and 0.5) or Li[Li_(1-3x)/3_Ni_x_Co_x_Mn_(2-3x)/3_]O_2_ (where *x* = 1/3, 0.2, and 0.13) were synthesized in identical conditions through a sol–gel method assisted by citric acid as chelating agent (Table 1), which confirms that Li_2_MnO_3_-rich electrode materials exhibit superior electrochemical performance compared to the conventional LiNi_1/3_Co_1/3_Mn_1/3_O_2_ electrode. These compositions were analyzed to determine their optimal configuration and to understand their extraordinary behavior. Structural properties were investigated using XRD and Raman spectroscopy, while the morphology and grain-size distribution of the samples were characterized by BET, SEM and HRTEM analyses. The electrochemical performance of the integrated Li- and Mn-rich compounds was evaluated through galvanostatic charge–discharge (GCD) cycling and electrochemical impedance spectroscopy (EIS). 

## 2. Materials and Methods

### 2.1. Materials Synthesis 

**Figure 1:**
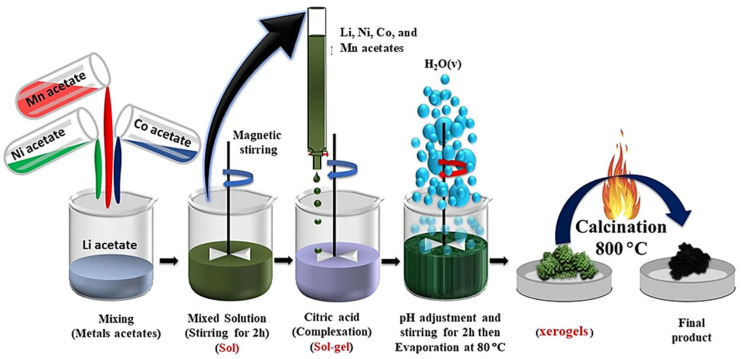
Schematic representation of the synthesis of integrated *y*Li_2_MnO_3_∙(1-*y*) LiNi_1/3_C_1/3_Mn_1/3_O_2_ cathode materials using the citric acid assisted sol–gel method (acetate route).

The oxide powders *y*Li_2_MnO_3_∙(1-*y*)LiNi_1/3_C_1/3_Mn_1/3_O_2_ (y = 0.0, 0.3 and 0.5) were prepared by the sol–gel method as illustrated in Figure 1. The precursor was prepared using acetate salts as the source of metal ions and citric acid as the chelating agent. Analytical-grade reagents (99.99%, Sigma-Aldrich)—including stoichiometric amounts of CH_3_COOLi∙2H_2_O, Ni(CH_3_COO)_2_∙4H_2_O, Co(CH_3_COO)_2_∙4H_2_O, and Mn(CH_3_COO)_2_∙4H_2_O—were used as starting materials for all the samples. The stoichiometric amounts of these salts were mixed and dissolved in deionized water under continuous stirring for 1 h. An excess of 7 mol% Li was introduced to account for potential mechanical and volatilization losses during subsequent transportation and calcination. The molar ratio of chelating agent (citric acid) to total metal ions was maintained for unity. Citric acid was carefully added step by step to the stirred aqueous solution of metal cations under a controlled pH concentration and temperature. The pH of the solution was adjusted to approximatively 7 using an alkaline solution of ammonium hydroxide, and the temperature was maintained at 80 °C. The resulting solution was stirred vigorously using a magnetic stirrer to facilitate evaporation, leading to the formation of a viscous transparent gel. With further evaporation, the gel gradually transformed into a xerogel. Next, the obtained xerogel was dried in an oven at 120 °C for 12 h. The resulting precursors were first calcined at 450 °C for 5 h. After cooling, they were ground into fine powders and then subjected to a second calcination at 800 °C for 20 h in air with intermittent grinding. This process yielded the final products. 

### 2.2. Materials’ Characterization

The phase and structure of the final product were analyzed by X-ray diffraction (XRD) using the Philips X’Pert apparatus equipped with a CuK_α_ X-ray source (λ = 1.54056 Å). Data were collected in the 2*θ* range 10–80° at a step of 0.05°. The obtained XRD patterns were refined using FULLPROF software (Toolbar Fullprof suit program (3.00), version June-2015) [19]. The surface morphology and composition of the fabricated samples were investigated by scanning electron microscopy using the ZEISS model ULTRA 55, equipped with an energy-dispersive X-ray spectrometer (EDX). HRTEM images were obtained using an electronic microscope, JEOL model JEM- 2010. The Brunauer–Emmett–Teller (BET) surface area and pore-size distribution of the synthesized samples were determined from N_2_-adsorption experiments using Belsorp max version 2.3.2. The BET surface area was calculated from adsorption isotherms ranging from 0.02 to 0.4 of relative pressures (*P*/*P*_0_). Raman scattering spectra were recorded using a Horiba micro-Raman spectrophotometer equipped with an optical microscope. The measurements were performed with a 633 nm He–Ne laser excitation line, using a step size of 1.6 cm^−1^ and an acquisition time of 30 s. A ×100 microscope objective was employed to focus the laser beam and collect scattered light, resulting in a laser spot with a diameter of approximately 1 μm. To prevent sample photo-decomposition, the laser power was kept low at 100 W cm^−2^. The wavenumber calibration was routinely verified using the 520 cm^−1^ Raman peak of a silicon crystal as a reference. 

Electrochemical tests were conducted using CR2025-type coin cells. The cathodes were fabricated by mixing 80 wt.% active material, 10 wt.% carbon black (as a conductive agent), and 10 wt.% polyvinylidenefluoride (PVDF) dissolved in N-methyl pyrrolidinone (NMP) to form a homogeneous slurry. The slurry was evenly coated onto aluminum foil (serving as the current collector) and dried at 80 °C for 2 h to remove the solvent. After drying, the foil was pressed to enhance adhesion and ensure uniform thickness. The electrode films were punched into disks with a diameter of approximately 10 mm and dried under vacuum at 80 °C for 12 h. The cathode loading was estimated to be around 2 mg cm^−2^. The cells were assembled using a lithium sheet as the counter electrode and Celgard 2500 or 2300 film as the separator. The electrolyte consisted of 1 mol L^−1^ LiPF_6_ in a mixture of ethylene carbonate (EC) and dimethyl carbonate (DMC) (1:1) (LP30, Merk, Rahway, NJ, USA). All assembly procedures were conducted at room temperature in a glove box under an argon atmosphere with moisture and oxygen levels maintained at ≤5 ppm. The galvanostatic charge–discharge curves were assessed using a potentiostat/galvanostat (VMP3 Bio-Logic) over a potential range of 2.0–4.8 V.

## 3. Results

### 3.1. Structural Investigations

**Figure 2:**
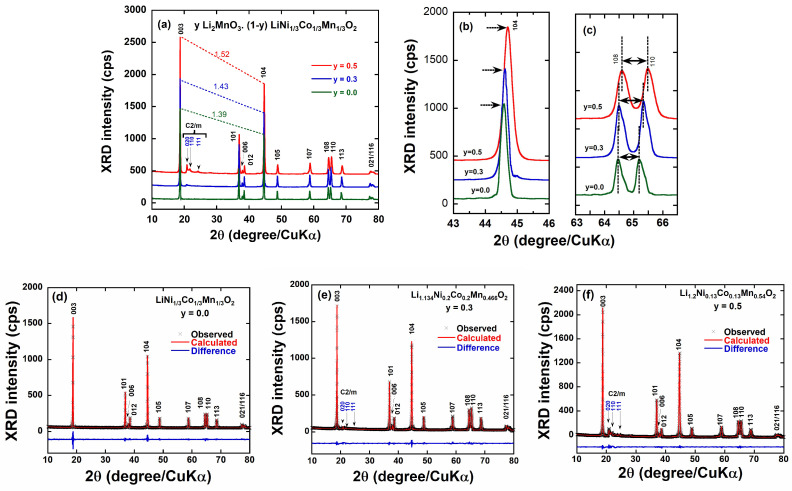
(**a**) X-ray diffraction (XRD) patterns of integrated layered cathode materials. (**b**) XRD reflections at ca. 2*θ* = 44.5°, (**c**) detailed XRD patterns in the 2*θ* range 63–67°, and (**d**–**f**) Rietveld refinements of the as-prepared *y*Li_2_MnO_3_∙(1-*y*)LiNi_1/3_C_1/3_Mn_1/3_O_2_ (0.0 ≤ *y* ≤ 0.5) prepared by the sol–gel method.

The X-ray diffraction patterns of the as-prepared *y*Li_2_MnO_3_∙(1-*y*) LiNi_1/3_Co_1/3_Mn_1/3_O_2_ powders are shown in Figure 2a, while magnified diffractograms in the 2*θ* range 43–46° and 63–67° are presented in Figure 2b and Figure 2c, respectively. All diffractograms exhibit the characteristic patterns of the rhombohedral α-NaFeO_2_ layered structure with the *R*-3*m* space group (standard card JCPDS 82-1495) [20]. For *y* = 0.0, no secondary phases are observed, and the well-resolved splitting of the (006)/(012) and(108)/(110) diffraction doublets confirms the well-ordered crystallized layered structure. The Li-rich oxides (*y* = 0.3 and 0.5) display the same diffraction pattern as the parent sample, with additional weak reflection peaks appearing in the 2*θ*-range 20–25° (the intensity increasing with *y*). These peaks can be indexed to the (020), (110), and (111) lattice planes of Li_2_MnO_3_ with monoclinic *C*2/*m* symmetry, indicating the existence of a superlattice structure within Li_2_MnO_3_ [21]. These reflections arise due to Li^+^ ions located in the transition metal layers [22]. As expected, the integral intensity of these peaks strongly correlates with the Li_2_MnO_3_ content, increasing significantly with higher Li_2_MnO_3_ concentrations in the integrated *y*Li_2_MnO_3_∙(1-*y*)LiNi_1/3_C_1/3_Mn_1/3_O_2_ series. Upon closer examination of the 2*θ* region around 44.5° (Figure 2b), a slight shift in the Bragg peak corresponding to the (104) plane is observed toward higher 2*θ* values with increasing lithium content, likely due to changes in the lattice parameters. 

The clear separation of diffraction doublets, such as the (006)/(012) and (108)/(110) peaks, in Li-rich layered cathode materials provides valuable insights into the material’s structural and electrochemical properties. To be more specific, the separation of these doublets is a hallmark of a well-ordered layered structure (space group *R*-3*m* for NCM or *C*2/*m* for Li_2_MnO_3_). A larger separation indicates a higher degree of cation ordering between the lithium and transition metal layers, which correlates with better structural stability during cycling. Clear separation suggests a minimal mixing of cations between the transition metal layers and lithium layers (i.e., a reduced cation disorder). Cation disorder can hinder lithium-ion mobility and degrade electrochemical performance. Well-separated peaks also indicate distinct lithium and transition metal layers, which provide defined pathways for lithium-ion diffusion. As a consequence, materials with better-separated diffraction peaks exhibit a higher initial capacity, due to reduced cation disorder and improved activation of the Li_2_MnO_3_ phase. They also exhibit better cycling stability, as a more ordered structure resists phase transitions and mechanical strain during repeated lithium intercalation/deintercalation.

Further analysis of the XRD patterns for *y*Li_2_MnO_3_∙(1-*y*)LiNi_1/3_Co_1/3_Mn_1/3_O_2_ (0.0 ≤ *y* ≤ 0.5) was conducted using the Rietveld refinement via the Fullprof program. It was assumed that the integration of rhombohedral and monoclinic phases at the atomic level accounts for all the diffraction peaks in the patterns. Therefore, the procedure included adjusting the occupancy ratios of transition metal ions (Ni, Mn, and Co) at the 3*b* site, as well as the Li^+^ ions and a small fraction of Ni^2+^cations at the 3*a* site. Additionally, the Mn/Li ratio at sites 4*g* and 2*b* in the Li_2_MnO_3_ crystal structure was refined [15,23]. The refined XRD spectra are presented in Figure 2d–f, with the corresponding results summarized in Table 2. In these figures, the black cross marks represent experimental data, while the red solid lines are the calculated spectra. The minimal difference between calculated and experimental diffractograms highlights the high quality of the fitting process. This is further supported by the low values of residual and reliability parameters (*R*_p_, *R*_w_, and *χ*_2_) obtained from the Rietveld refinement, which confirm the successful identification of the as-prepared samples even in the presence of both rhombohedral and monoclinic phases. These results validate the structural model. In the Rietveld refinement, the phase fraction was determined with an uncertainty of 0.1%, achieved by minimizing the difference between the experimental and calculated diffractograms.

**Figure 3:**
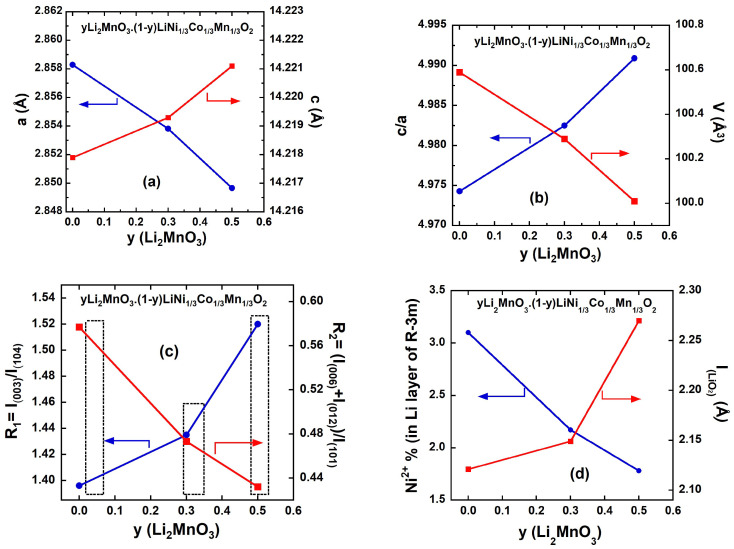
Structural properties of *y*Li_2_MnO_3_∙(1-*y*) LiNi_1/3_Co_1/3_Mn_1/3_O_2_ samples as a function of *y*(Li_2_MnO_3_). (**a**) Evolution of the refined lattice parameters *a*_hex_ and *c*_hex_ using an hexagonal system. (**b**) Variation in the c/a ration and cell volume. (**c**) Variations in the R-factors. (**d**) Variation in the amount of Ni^2+^ in the Li site and interslab thickness (*I*_(LiO2)_).

Figure 3a–d presents the structural analysis of the *y*Li_2_MnO_3_∙(1-*y*)LiNi_1/3_C_1/3_Mn_1/3_O_2_ samples as a function of y content. The lattice parameters (*a* and *c*) and the *c*/*a* ratio are summarized in Table 2. Across a wide composition range, the solid lines in Figure 3a indicate that the solid solution obeys the Vegard law. As the y content increases, slight changes in the lattice parameters are observed: the *a*-parameter is reduced and *c*-parameter increases. These changes are attributed to differences in ionic radii of Mn^4+^, Ni^2+^, and Co^3+^cations. An increase in the Li_2_MnO_3_ phase from 0.0 to 0.5 (corresponding to a reduction in the L*M*O_2_ phase) necessitates a higher Mn^4+^ content to maintain the charge balance. This results in a reduction in the unit cell volume by approximately 0.6 % (Figure 3b), as the ionic radius of Mn^4+^ (*r*_(Mn4+)_ = 0.53 Å) is smaller than that of Ni^2+^ (*r*_(Ni2+)_ = 0.69 Å) and Co^3+^ (*r*_(Co3+)_ = 0.545 Å) [24].The intensity ratios of the specific Bragg reflections, *R*_1_ = I_(003)_/I_(104)_ and *R*_2_= (I_(006)_+I_012_)/I_(101)_, which are associated with the rhombohedral phase are commonly used to assess the degree of cation mixing in the layered lattice. Additionally, the *c*/*a* ratio serves as an indicator of the deviation from the rock salt structure. The variations in R-factors for the hexagonal lattice are shown in Figure 3c. A higher *R*_1_ (greater than 1.2) and a lower *R*_2_ value (less than 1.0) indicate low cation mixing and improved hexagonal ordering [25,26]. When *R*_1_ > 1.2, the system has a more ordered arrangement, with fewer defects or disordered cation mixing. It means that the cations are occupying their designated sites with more precision, contributing to lower cation mixing. *R*_2_ < 1.0 indicates a more regular or symmetric arrangement of cations, implying better structural ordering within the crystal lattice. A value of *R*_2_ less than 1.0 suggests reduced disorder and the absence of significant cation mixing, which allows for more uniform and efficient bonding, further enhancing the material’s structural integrity and symmetry. Low cation mixing (reflected by a high *R*_1_ and low *R*_2_) generally leads to better ordering in hexagonal systems, as the positions of cations are more predictable, allowing the crystal lattice to adopt an idealized, low-energy configuration. These factors contribute to a more stable, ordered material with improved properties, such as enhanced conductivity or structural stability.

As seen in Figure 3c, all the samples exhibit *R*_1_ values greater than 1.2 and *R*_2_ values below 0.5, confirming their well-ordered structure and minimal cation mixing. This observation provides further evidence that all the synthesized *y*Li_2_MnO_3_∙(1-*y*)LiNi_1/3_Co_1/3_Mn_1/3_O_2_ oxides consist of integrated Li_2_MnO_3_/LiMO_2_-like components with a characteristic layered structure. For example, in Table 2, the more reliable Rietveld refinement results indicate that introducing LiMn_2_O_3_ at any proportion reduces the anti-site Ni^2+^–Li concentration defects. Specifically, the introduction of 50% LiMn_2_O_3_ reduces the Ni^2+^ ions at the 3*a* site by approximately 43% (Figure 3d). The mitigation of cationic-mixing mitigation is by the higher *c*/*a* ratio; the highest *R*_1_ and lowest *R*_2_ values observed for the Li_1.2_Ni_0.13_Co_0.13_Mn_0.54_O_2_ (at *y* = 0.5) sample.

**Figure 4:**
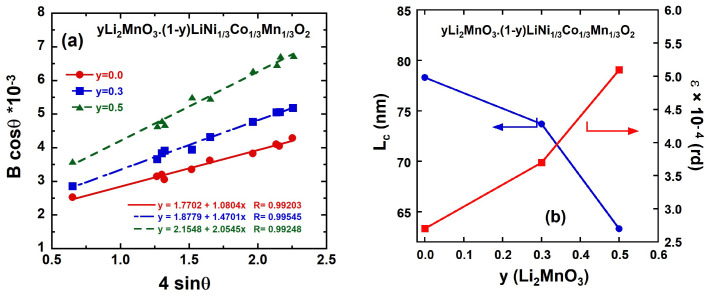
(**a**) Analysis of microstrain from the full-width B at half-maximum of the XRD peaks according to Equation (1) (**b**) Evolution of the crystallite size and strain field (see Equation (1)) as a function of Li_2_MnO_3_ content (*y*).

To gain deeper insights into the structural properties of integrated *y*Li_2_MnO_3_∙(1-*y*)LiNi_1/3_C_1/3_Mn_1/3_O_2_ oxides, the TM slab thickness (*S*_(MO2)_) and interslab thickness (*I*_(LiO2)_) were calculated using the hexagonal cell parameter *c*_hex_ and the atomic coordinate of oxygen ions (*z*_ox_) (Table 2). As the Li_2_MnO_3_ content increases from 0.0 to 0.5, *S*_(MO2)_ decreases by 5.65%, while *I*_(LiO2)_ increases by 7.02%. This behavior is attributed to the reduced amount of Ni^2+^ ions in the interslab space with a higher LiMn_2_O_3_ content, resulting in weaker screening between the oxygen layers in the interslab region. The increase in *I*_(LiO2)_ can facilitate the rapid diffusion of lithium ions, thereby enhancing electrochemical performance, as a larger *I*_(LiO2)_ improves the diffusion coefficient of Li^+^ ions. Additionally, the reduction in *S*_(MO2)_ contributes to improved structural stability, effectively mitigating the TM dissolution. [27]. Additional insights into the structural properties can be derived from the broadening of diffraction peaks, which serves as an indicator not only of the crystallinity of the as-prepared *y*Li_2_MnO_3_∙(1-*y*)LiNi_1/3_Co_1/3_Mn_1/3_O_2_ powders but also of the homogeneous distribution of cations within the structure. The microstrain (*ε*) of the particles was determined using the Williamson–Hall equation [28]:

B_hkl_ cos θ_hkl_ = (Kλ/L_c_) + 4 ε sin θ_hkl_ (1)

where λ is the X-ray wavelength, K is the shape factor, B_hkl_ is the line broadening of a Bragg reflection (hkl), and L_c_ is the effective crystallite size. The first member (B_hkl_ cos_θ__hkl_) is reported as a function of 4sinθ_hkl_ in (Figure 4a) for the *y*Li_2_MnO_3_∙(1-*y*)LiNi_1/3_Co_1/3_Mn_1/3_O_2_ samples. The plots are well fitted by straight lines, in agreement with Equation (1). The microstrain ε was estimated from the slope of the lines, while the crystallite size *L*_c_ was determined from the intercept with the vertical axis. The resulting values are summarized in Table 2. As shown in Figure 4b, the microstrain *ε* increases significantly with *y*. In contrast, the crystallite size values remain within a narrow range of 63.3 ≤ L_c_ ≤ 78.3 nm (Figure 4b and Table 2), indicating that the addition of Li_2_MnO_3_ has no notable effect on the coherence length. The nearly identical L_c_ values highlight the uniformity of the synthesis process, demonstrating that the use of citric acid effectively preserves the layered framework, even with the Li_2_MnO_3_ increased up to 50%.

### 3.2. Morphological Characterization

**Figure 5:**
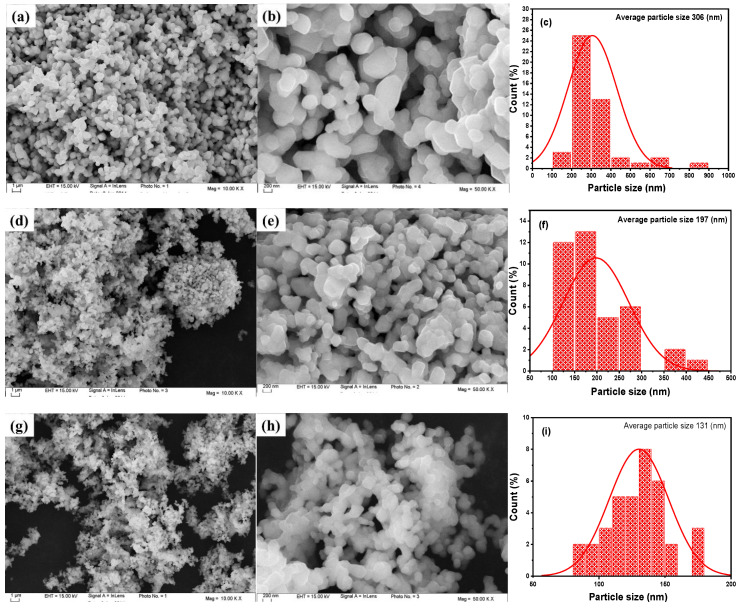
SEM images at magnifications of 10 k and 50 k and particle-size distribution of *y*Li_2_MnO_3_∙(1-*y*)LiNi_1/3_Co_1/3_Mn_1/3_O_2_ powders: (**a**–**c**) for *y* = 0.0 (LiNi_1/3_Co_1/3_Mn_1/3_O_2_), (**d**–**f**) for *y* = 0.3 (Li_1.134_Ni_0.2_Co_0.2_Mn_0.466_O_2_), and (**g**–**i**) for *y* = 0.5 (Li_1.2_Ni_0.13_Co_0.13_Mn_0.54_O_2_).

**Figure 6:**
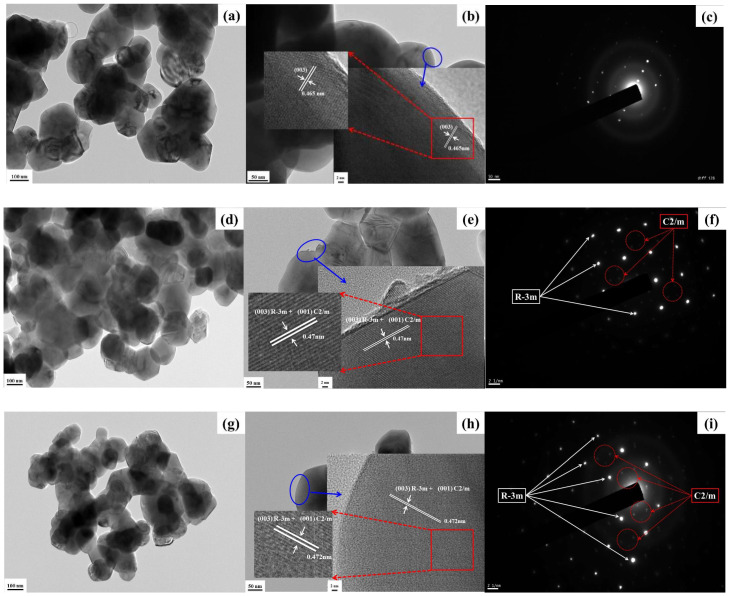
TEM (**a**,**d**,**g**), HRTEM (**b**,**e**,**h**), and SAED (**c**,**f**,**i**) images of *y*Li_2_MnO_3_∙(1-*y*) LiNi_1/3_Co_1/3_Mn_1/3_O_2_ powders: (**a**–**c**) for *y* = 0.0 (LiNi_1/3_Co_1/3_Mn_1/3_O_2_), (**d**–**f**) for *y* = 0.3 (Li_1.134_Ni_0.2_Co_0.2_Mn_0.466_O_2_), and (**g**–**i**) for *y* = 0.5 (Li_1.2_Ni_0.13_Co_0.13_Mn_0.54_O_2_).

It is well established that particle size, surface morphology, and particle distribution are crucial factors influencing the performance of Li-ion batteries. Electron microscopy analyses, including SEM, TEM, and HRTEM, were conducted for the *y*Li_2_MnO_3_∙(1-*y*)LiNi_1/3_Co_1/3_Mn_1/3_O_2_ (*y* = 0.0, 0.3, 0.5) powders, and the results are presented in Figure 5 and Figure 6, respectively. The SEM images reveal that all the powders exhibit regular particles with a similar morphology, consisting of uniform, spherical-shaped primary particles. The pristine LiNi_1/3_Co_1/3_Mn_1/3_O_2_ powder (Figure 5a,b) shows the largest grain size, with an average diameter of 200–400 nm, along with partial agglomeration. As the *y*Li_2_MnO_3_ content increases (equivalent to an increase in Li concentration), the grain size decreases, and the size distribution becomes narrower. This reduction in particle size can be attributed to the increased Li content, which not only enhances phase stability but also promotes a more uniform structure. Additionally, the increased Li concentration reduces the crystal surface energy, which can induce atomic-level changes in the crystal structure, leading to the formation of defects or the nucleation of smaller particles during synthesis. These effects help control particle growth, promoting the formation of smaller, more uniform particles. For example, the Li_1.2_Ni_0.13_Co_0.13_Mn_0.54_O_2_ powder (Figure 5g,h) exhibits particles with a thickness of approximately 130 nm, which aligns with the XRD results. The effect of Li concentrations on the grain size of Li-rich oxides is consistent with findings from our previous work [29].

Figure 6 presents TEM images of the *y*Li_2_MnO_3_∙(1-*y*)LiNi_1/3_Co_1/3_Mn_1/3_O_2_ powders, showing homogeneous, sphere-like particles with sizes in the range 100–200 nm. The corresponding HRTEM images (Figure 6b,e,h) reveal distinct lattice fringes with a *d*-spacing of approximately 0.47 nm, which aligns well with the inter-planar distance of the (003)_hex_ of the Li*M*O_2_ plane and/or the (001)_mon_ plane of Li_2_MnO_3_, reflecting remarkable structural compatibility between the two phases [5,30,31]. This structural similarity makes it challenging to differentiate between the two layered structures. All the samples exhibit clearly defined fringes in the HRTEM images, indicating good crystallinity. A comparison of TEM images confirms that the size of particles decreases with an increasing *y* (i.e., Li_2_MnO_3_ content), consistent with the XRD results. The selected area electron diffraction (SAED) patterns of the *y*Li_2_MnO_3_∙(1-*y*)LiNi_1/3_Co_1/3_Mn_1/3_O_2_ powders are shown in Figure 6c,f,i. The lattice patterns confirm the highly crystalline nature of all the samples. At *y* = 0.0 (Figure 6c), the SAED pattern shows only one type of reflection, corresponding to the rhombohedral *R*-3*m* phase. With an increasing *y*, (Figure 6f,i), new reflections emerge, and the SAED patterns predominantly consist of two types of reflections: strong fundamental reflections (marked as solid white arrows) indicating the presence of the rhombohedral *R*-3*m* phase, resulting from the random distribution of cations in the TM layer without any long-range ordering. This evolution in the SAED patterns with increasing Li_2_MnO_3_ content reflects the coexistence of Li_2_MnO_3_ and Li*M*O_2_-like components in the material. Weak triplet reflections appear between two fundamental reflections (marked with dotted red arrows). The the existence of these triplet dark spots indicates the presence of monoclinic Li_2_MnO_3_-like domains, and these reflections highlight the ordering of lithium ions alongside cations in the TM layers [32,33,34]. The incorporation of lithium ions into the TM layer establishes long-range ordering within the unit cell. The presence of well-resolved lattice fringes further confirms the excellent crystallinity of the samples. The above results confirm the existence of a Li_2_MnO_3_-LiNi_1/3_C_1/3_Mn_1/3_O_2_ solid solution and indicates the structural compatibility between the *R*-3*m* and *C*2/*m* phase sharing the same lattice.

**Figure 7:**
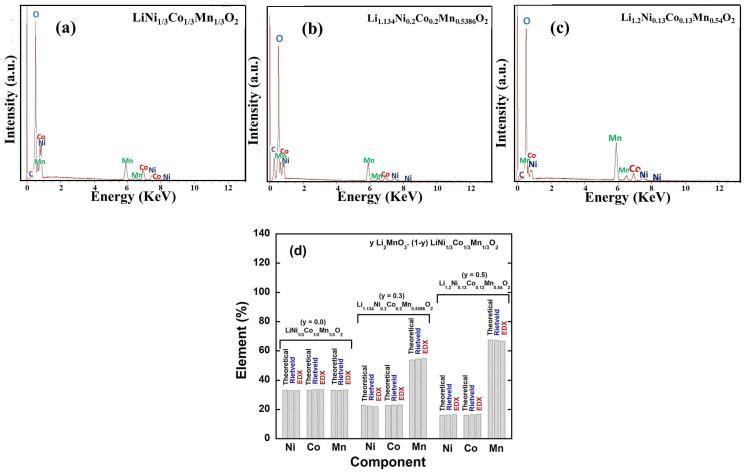
(**a**–**c**) EDX spectra and (**d**) comparison between theoretical and experimental values for 3D elements of prepared *y*Li_2_MnO_3_∙(1-*y*) LiNi_1/3_C_1/3_Mn_1/3_O_2_ (0.0 ≤ *y* ≤ 0.5) powders.

In addition to Rietveld refinement, energy-dispersive X-ray spectroscopy (EDX) experiments were conducted to verify the chemical composition of the *y*Li_2_MnO_3_∙(1-*y*)LiNi_1/3_C_1/3_Mn_1/3_O_2_ powders. The EDX spectra of the synthesized oxides are presented in Figure 7a–c. Due to lithium’s extremely weak scattering factor and low X-ray fluorescence yield, it cannot be detected through the Rietveld refinement of XRD data or EDX analysis. Apart from the peaks corresponding to Ni, Co, and Mn, as well as the characteristic peak of the carbon foil used for SEM experiments, no peaks for any elements were observed. This confirms the absence of any impurities in all the samples. Table 3 presents the theoretical and experimental concentrations of Ni, Co, and Mn (in atomic percentage) for the *y*Li_2_MnO_3_∙(1-*y*)LiNi_1/3_C_1/3_Mn_1/3_O_2_ samples, as determined from Rietveld refinement and EDX analysis. Figure 7d shows the composition of 3d elements Ni, Co, and Mn. The Mn content is obviously higher tnan Ni and Co, and the content of Ni and Co is quite equal in the as-prepared *y*Li_2_MnO_3_∙(1-*y*) LiNi_1/3_C_1/3_Mn_1/3_O_2_ (*y* = 0.3, 0.5) powders. The deviation in the measured values of Ni, Co, and Mn from their theoretical content does not exceed 1.0%, indicating a satisfactory agreement between the nominal formula and the experimental results. The consistency with the ideal stoichiometry value further validates the phase analysis results derived from the XRD data. 

**Figure 8:**
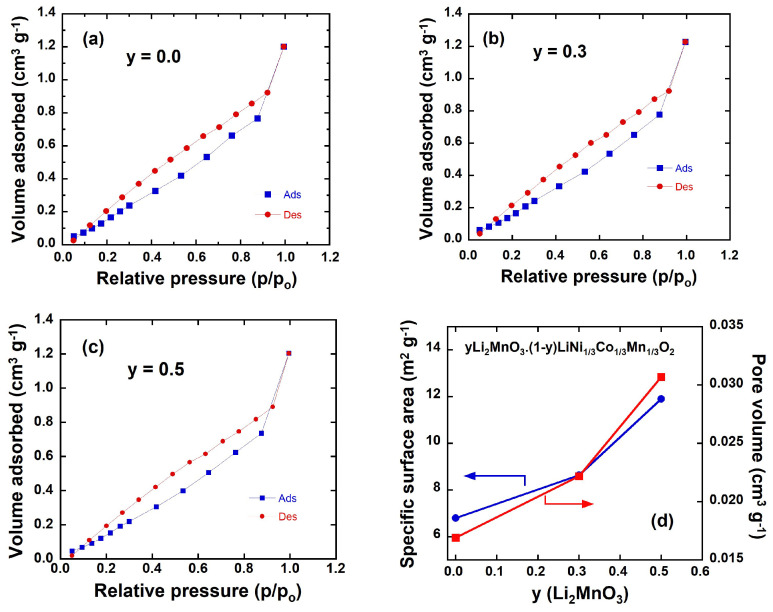
(**a**–**c**) Nitrogen adsorption–desorption isotherms for *y*Li_2_MnO_3_∙(1-*y*)LiNi_1/3_C_1/3_Mn_1/3_O_2_ (0.0 ≤ *y* ≤ 0.5) powders. (**d**) Variation in specific surface area and pore volume as function of *y*(Li_2_MnO_3_).

Figure 8a–c display the nitrogen adsorption–desorption isotherms of the *y*Li_2_MnO_3_∙(1-*y*)LiNi_1/3_C_1/3_Mn_1/3_O_2_ powders. All three samples exhibit similar isotherm shapes, featuring a hysteresis loop indicative of a hierarchical nanoporous structure [35]. The isotherms show an increase with a rising *p*/*p*_0_, forming a hysteresis loop up to *p*/*p*_0_ ≈ 0.92, which, according to the IUPAC classification, corresponds to a type IV isotherm with an H3 hysteresis loop [36]. The pore structure, calculated using the Barrett–Joyner–Halenda (BJH) model, reflects the interconnecting voids between randomly packed nanoparticles. 

As summarized in Table 4, the BJH pore-size distribution confirms the nanopore nature of all the samples. Based on the results shown in Figure 8d and the data listed in Table 4, the BET specific surface area (*S*_BET_) and pore volume of pristine LiNi_1/3_C_1/3_Mn_1/3_O_2_ are 6.8 m^2^ g^−1^ and 0.0169 m^3^ g^−1^, respectively. These values increase with the rising *y*(Li_2_MnO_3_) content, which can be attributed to the presence of two distinct solid phases. Each phase inhibits the growth of the other, resulting in composite powders with smaller average crystal sizes and higher surface areas, even after high-temperature post-processing [27], i.e., a second calcination at 800 °C for 20 h in air with intermittent grinding. Conversely, the average particle diameter L_BET_ (in nm) decreases as the *y*(Li_2_MnO_3_) content increases, as shown in Table 4. This particle diameter (L_BET_) can be estimated from BET measurements using the relation [37]:

(2) $$L_{BET}=\frac{6000}{S_{BET}d},$$

where L_BET_ is expressed in nm, S_BET_ is the specific surface area (in m^2^ g*^−^*^1^), and d is the gravimetric density (*d* = 4.78, 4.42, and 4.25 g cm^−3^ for *y* = 0.0, 0.3, and 0.5, respectively). The L_BET_ values align closely with the particle sizes L_SEM_ determined from SEM patterns. Additionally, it is observed that as the amount of Li_2_MnO_3_ also rises, this enhanced porosity improves wettability, facilitating better the penetration of the electrolyte, and consequently shortening the diffusion paths within the cathode material.

### 3.3. Vibrational Properties

**Figure 9:**
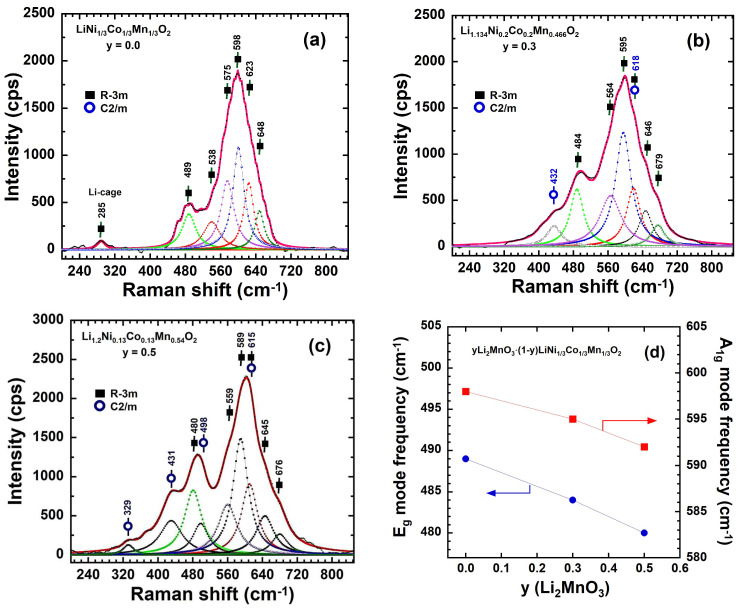
Raman scattering spectra of integrated *y*Li_2_MnO_3_∙(1-*y*)LiNi_1/3_C_1/3_Mn_1/3_O_2_ powders: (**a**) y = 0.0, (**b**) *y* = 0.3, (**c**) *y* = 0.5. (**d**) Frequency shift in the *A*_1g_ and *E*_g_ modes against the composition.

Raman scattering (RS) spectroscopy was employed to investigate the local structure, surface state, and composition of the as-prepared samples [38]. This technique serves as a surface-sensitive probe, capable of analyzing the short-range oxygen coordination around the cations in oxide frameworks. The Raman scattering spectra of the *y*Li_2_MnO_3_∙(1-*y*)LiNi_1/3_C_1/3_Mn_1/3_O_2_ powders are shown in Figure 9a–c. Overall, the spectra display features characteristic of layered Li*M*O_2_ (*M* = Ni, Mn, or Co) and Li_2_MnO_3_. For the rhombohedral Li*M*O_2_ oxide with the *R*-3*m* (*D*_3d_^5^) space group (spectroscopic symmetry), two Raman-active modes (*A*_1g_+*E*_g_) are predicted. These arise from *M*–O stretching (around 480 cm^−1^) and O–M–O bending (around 600 cm^−1^) vibrations, respectively. Each band reflects a superposition of contributions from the three transition metal ions, resulting in three *A*_1g_ and three *E*_g_ modes. Similarly, for the monoclinic Li_2_MnO_3_ oxide with a *C2/m* space group (*C*_2h_^3^ spectroscopic symmetry), six Raman-active modes (4*A*_g_+2*B*_g_) are predicted, giving rise to nine peaks located at 248, 308, 332,339, 413, 438, 439, 568, and 612 cm^−1^ [39].

The Raman spectra of the prepared samples (0.0 ≤ *y* ≤ 0.5) are shown in Figure 9a–c. The Raman spectrum of the pristine LiNi_1/3_Co_1/3_Mn_1/3_O_2_ exhibits two broad bands centered at approximately 489 and 599 cm^−1^, resulting from the overlap of the three *A*_1g_ and *E*_g_ modes (Figure 9a). To analyze these overlapping bands, the spectra were deconvoluted using a set of three Lorentzian-shaped individual bands, achieving the best fit. For Li-rich compounds (Figure 9b,c), the spectra prominently feature the two dominant *A*_1g_ and *E*_g_ modes associated with the *R*-3*m* phase, along with additional weak vibrational bands corresponding to the monoclinic Li_2_MnO_3_ phase. These observations are consistent with the mixed-phase nature of the Li-rich samples [11]. In the Raman spectrum of Li_1.134_Ni_0.2_Co_0.2_Mn_0.467_O_2_ **(**Figure 9b), a small additional vibration band at 428 cm^−1^, corresponding to the *A*_g_ mode, was observed. In contrast, the Raman spectrum of Li_1.2_Ni_0.13_Co_0.13_Mn_0.54_O_2_ (Figure 9c) reveals not only the 428 cm^−1^ band but also additional vibration bands at 498 cm^−1^, a shoulder at 565 cm^−1^, and another band at 329 cm^−1^, all derived from the *B*_g_ modes. These extra peaks are associated with the monoclinic Li_2_MnO_3_ phase. However, they were barely detectable in the XRD spectrum, as this additional phase was poorly crystallized. XRD is sensitive to well-ordered structures with coherence lengths significantly larger than the lattice parameters, making it challenging to identify ill-crystallized phases. Raman spectroscopy, with its sensitivity to local order at the molecular scale, is an ideal tool for detecting the presence of this additional phase. For Li-rich samples, the *A*_1g_ peak around 600 cm^−1^ appears relatively sharp and without any splitting. Moreover, as shown in Figure 9d, increasing the *y*(Li_2_MnO_3_) content results in a shift in the *A*_1g_ and *E*_g_ modes to lower frequencies. This shift can be attributed to an increase in the inner slab bond covalency in the LiNi_1/3_C_1/3_Mn_1/3_O_2_ structure, which is consistent with the observed decrease in metal–metal intralayer distances (as reported in Table 2 from the XRD data). These findings suggest that the Li_2_MnO_3_ region and the Li*M*O_2_ region are well integrated, forming a homogeneous composite structure [40,41,42]. 

### 3.4. Electrochemical Properties

**Figure 10:**
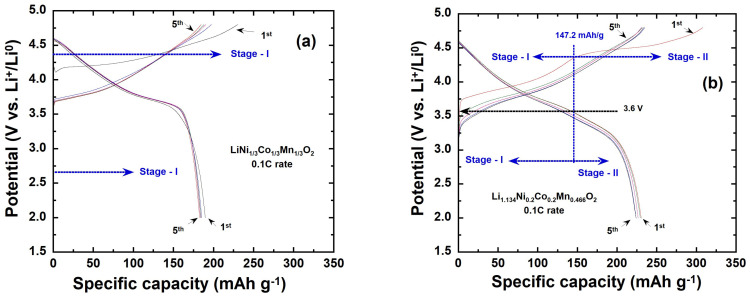

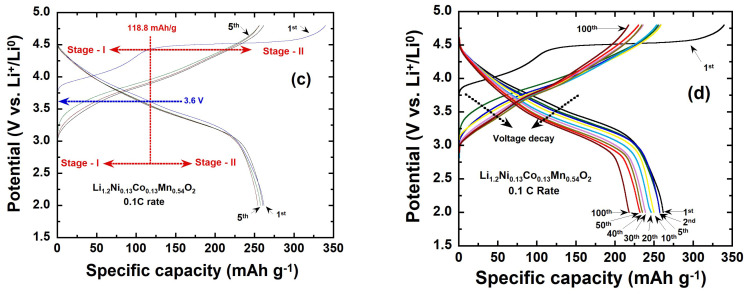
Galvanostatic charge–discharge capacity curves recorded at C/10 rate in potential range 2.0–4.8 V vs. Li^+^/Li for (**a**) LiNi_1/3_Co_1/3_Mn_1/3_O_2_, (**b**) Li_1.134_Ni_0.2_Co_0.2_Mn_0.466_O, (**c**) Li_1.2_Ni_0.13_Co_0.13_Mn_0.54_O_2_ until 5 cycles, (**d**) Li_1.2_Ni_0.13_Co_0.13_Mn_0.54_O_2_ over 100 cycles.

The galvanostatic charge–discharge curves for the first five cycles of the *y*Li_2_MnO_3_∙(1-*y*)LiNi_1/3_C_1/3_Mn_1/3_O_2_ (*y* = 0.0, 0.3, and 0.5) electrode materials and the first 100 cycles for Li_1.2_Ni_0.13_Co_0.13_Mn_0.54_O_2_ (*y* = 0.5) at a C/10 rate, within a voltage range of 2.0 V to 4.8 V, are presented in Figure 10a–d. With increasing lithium content, the sloping profile of the first charge curve becomes more pronounced, and a distinct plateau appears. This behavior is attributed to phase transformations and variations in site occupancy energy, as the reaction voltage is influenced by the lithium chemical potential. The shape and evolution of the charge–discharge curves after the first activation cycle align with the findings reported in the literature [29,43,44,45,46]. The capacity curves can be divided into two distinct stages: (I) from the open-circuit potential (OCP) to below 4.5 V and (II) above 4.5 V vs. Li^+^/Li (as shown in Figure 10). During the initial charge process, Li^+^ ions are deintercalated from the *R*-3*m* phase (stage I, Figure 10b,c), where the sloping voltage corresponds to the oxidation of Co^3+^ and Ni^2+^ (Equation (3)). This is followed by the activation of Li_2_MnO_3_ (stage II, Figure 10b,c), as Li^+^ is deintercalated from the *C*2/*m* phase. The voltage plateau at 4.5 V is attributed to oxygen loss accompanied by lithium removal, resulting in the evolution of O_2_ gas [47] and structural reorganization (Equation (4)). This activation process not only contributes to the structural rearrangement but also plays a crucial role in delivering additional capacity [48,49]:

*y*Li_2_Mn^4+^O_3_·(1-*y*)LiNi^2+^_1/3_Co^3+^_1/3_Mn^4+^_1/3_O_2_ → *y*Li_2_Mn^4+^O_3_∙(1-*y*) Ni^4+^_1/3_Co^4+^_1/3_Mn^4+^_1/3_O_2_+ (1-*y*)Li^+^ + (1-*y*)e^−^ (3)

*y*Li_2_Mn^4+^O_3_·(1-*y*) Ni^4+^_1/3_Co^4+^_1/3_Mn^4+^_1/3_O_2_ → *y*Mn^4+^O_2_·(1-*y*) Ni^4+^_1/3_Co^4+^_1/3_Mn^4+^_1/3_O_2_+ 2*y*Li^+^+ 0.5*y*O_2_ + 2*y*e^−^ (4)

During the first discharge process, Li^+^ ions are initially intercalated into the *M*O_2_ phase (stage I, Figure 10b,c). This is followed by reinsertion into the MnO_2_ (stage II, Figure 10b,c):

*y*Mn^4+^O_2_·(1-*y*) Ni^4+^_1/3_Co^4+^_1/3_Mn^4+^_1/3_O_2_+ Li^+^+e^−^ →*y* LiMn^3+^O_2_ (1-*y*)LiNi^2+^_1/3_Co^3+^_1/3_Mn^4+^_1/3_O_2_ (5)

According to Equations (3) and (5), the theoretical capacities of the LiNi_1/3_Co_1/3_Mn_1/3_O_2_ and Li_2_MnO_3_ components were calculated for comparison with the experimental data and are summarized in Table 5. During the first charge process (Figure 10a–c), it is widely accepted that 4.5V is a critical potential. Below this voltage, the capacity is primarily attributed to LiNi_1/3_C_1/3_Mn_1/3_O_2_, while above 4.5 V the capacity is associated with the activation of the Li_2_MnO_3_ phase. This distinction underscores the dual contribution of these components to the overall electrochemical performance. For the first discharge, a critical voltage of approximately 3.6 V is identified [50]. Above this voltage, the capacity is primarily contributed by the LiNi_1/3_Co_1/3_Mn_1/3_O_2_ component, while below this voltage, the capacity is attributed to LiMnO_2_, which is likely the product of Li_2_MnO_3_ after the first cycle (see Equation (5)). It is observed that the length of the charge plateau and consequently the charge capacities below 4.5 V—corresponding to the extraction of Li^+^ ions from the active LiNi_1/3_Co_1/3_Mn_1/3_O_2_ phase (the delithiation of the *R*-3*m* phase)—diminish as the Ni and Co contents decrease. This trend is consistent with an increasing Li content in the structure and aligns with the theoretical values presented in Table 5. This behavior reflects the balance between lithium-rich phases and transition metal contributions in determining the electrochemical characteristics. 

According to the data summarized in Table 5, together with the complete extraction of lithium ions, it can be seen that the practical capacity corresponding to the oxidation of Ni^2+^/Ni^4+^ and Co^3+^/Co^4+^ (below 4.5 V) are close to their theoretical values (predicted by Equation (3)) as the value of Li content increases. However, the Li_2_MnO_3_ phase cannot be oxidized, since manganese is already in the Mn^4+^ valence state. During the first discharge, lithium insertion occurs at around 4.4 V, reducing Ni^4+^ to Ni^2+^ and Co^3+^ (up to 3.6 V). This is followed by intercalation into the layered MnO_2_ component, reducing Mn^4+^ to Mn^3+^, as the voltage decreases to 2 V. For Li_2_MnO_3_-rich composites, a high initial discharge capacity is observed, which increases and approaches its theoretical values with rising lithium content. This indicates the effective integration of the Li_2_MnO_3_ phase into the 2D layered LiNi_1/3_Co_1/3_Mn_1/3_O_2_ structure, enhancing the overall capacity [51,52]. It is worth noting that the Li ions extracted during the first charge cannot fully reintegrate into the lattice structure during the first discharge. This is because not all oxidized oxygen can be reduced in the process, leading to a high irreversible capacity in the first cycle, as illustrated in Table 5.

In the second cycle, a voltage plateau around 4.5 V observed in the charge curve—a characteristic feature of Li-rich layered materials—disappears. This indicates that the activation of the Li_2_MnO_3_ phase is complete, accompanied by an irreversible structural change after the first charge. The capacities obtained during the first discharge at a 0.1C rate are 188, 230, and 262 mAh g^−1^ for *y* = 0.0, 0.3, and 0.5, respectively. Additionally, the initial Coulombic efficiencies are calculated to be 81.5%, 74.5%, and 76.7%, for *y* = 0.0, 0.3, and 0.5, respectively. These results highlight the influence of lithium content on both capacity and efficiency, with a higher Li_2_MnO_3_ content contributing to an increased capacity but slightly reduced initial Coulombic efficiency. The capacity loss observed in the first cycle is ascribed to the irreversible removal of Li_2_O from the Li_2_MnO_3_ region in the Li-rich samples, as well as side reactions with the electrolyte at a high operating voltage [53,54,55]. By the second cycle, the discharge capacities improve to 176, 228, and 256 mAhg^−1^ for *y* = 0.0, *y* = 0.3, and *y* = 0.5, respectively, with corresponding Coulombic efficiencies of 89.3, 96.6, and 98.1%. For the Li_1.2_Ni_0.13_Co_0.13_Mn_0.54_O_2_ electrode (*y* = 0.5), the discharge curve shifts to lower voltage plateaus with successive cyclings, as shown in Figure 10d. This voltage decay is likely associated with the gradual transformation of the layered structure into a spinel-like structure during cycling [46]. Despite this, the Li_1.2_Ni_0.13_Co_0.13_Mn_0.54_O_2_ electrode demonstrates excellent cyclic stability. After 100 cycles, it retains a capacity of 219 mAh g^−1^, with a nearly 100% Coulombic efficiency and a capacity retention of 83.6%. This highlights its promising performance for long-term energy storage applications.

**Figure 11:**
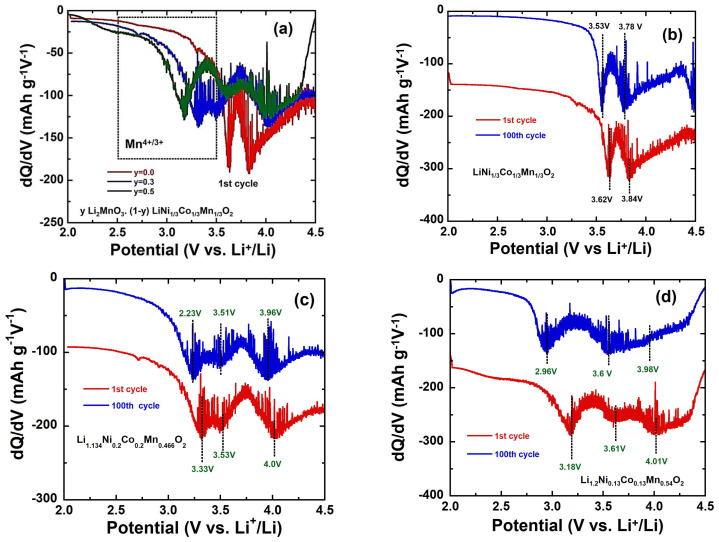
Differential capacity (−d*Q*/d*V*) vs. *V* plots for (**a**) *y*Li_2_MnO_3_∙(1-*y*) LiNi_1/3_Co_1/3_Mn_1/3_O_2_ electrodes at first cycle, (**b**) pristine LiNi_1/3_Co_1/3_Mn_1/3_O_2_, (**c**) Li_1.134_Ni_0.2_Co_0.2_Mn_0.466_O_2_, and (**d**) Li_1.2_Ni_0.13_Co_0.13_Mn_0.54_O_2_ at 1st and 100th cycles.

While the discharge–charge profiles demonstrate promising electrochemical performance for Li-rich cathode materials with increasing lithium content, the issues of voltage fade and differences in redox reaction potentials between the first cycle and the following cycles are better analyzed using differential (or incremental) capacity (d*Q*/d*V*) plots. Figure 11a presents the deferential d*Q*/d*V* versus *V* plots for the first discharge curves of *y*Li_2_MnO_3_∙(1-*y*)LiNi_1/3_Co_1/3_Mn_1/3_O_2_ electrodes with *y* = 0.0, 0.03, and 0.5. Figure 11b–d illustrate the differential capacity plots for the discharge curves of the same electrodes during the 1st and 100th cycles. The peaks in the -d*Q*/d*V* curves correspond to the pseudo-plateaus observed in the galvanostatic charge–discharge (GCD) profiles. These peaks provide insights into the redox processes and structural transformations occurring within the electrodes. By comparing the 1st and 100th cycle plots, the evolution of the redox reactions and the extent of voltage fade can be better understood. The differential capacity analysis highlights the dynamic changes in electrochemical behavior and helps identify the factors contributing to performance degradation over prolonged cycling. The differential -d*Q*/d*V* curve of pristine LiNi_1/3_Co_1/3_Mn_1/3_O_2_ (Figure 11a) confirms that the bump below 4.5V corresponds to the reduction of Ni^2+/3+/4+^ and/or CO^3+/4+^ during the insertion of Li^+^ ions into the layered framework. As the lithium content increases, these reduction peaks shift to higher voltages in the discharge profiles of Li-rich electrodes, reflecting the influence of the Li_2_MnO_3_ component on electrochemical behavior. A key distinction between pristine LiNi_1/3_Co_1/3_Mn_1/3_O_2_ and Li-rich electrodes is the emergence of a broad low-voltage cathodic peak below 3.5 V (as shown in Figure 11a). This peak is assigned to the reduction of Mn from the tetravalent state to a state slightly above trivalent (Equation (5)) in MnO_2_, which forms during the initial charge reaction (Equation (4)) [28]. This feature highlights the distinct redox processes and structural contributions brought about by the Li_2_MnO_3_ phase in Li-rich materials, enhancing their capacity but also introducing unique challenges, such as voltage fade. During cycling, the reduction reaction of Ni and/or Co ions become severely weakened, with their reduction potentials shifting to more negative values. Concurrently, the reduction reactions of manganese ions are enhanced, and their potentials also shift negatively. Consequently, capacity fading is primarily attributed to the reduced activity reduction of transition metals ions, while voltage decay arises from the negative shift in their reduction potentials. This is further compounded by the relative decrease in Ni and Co reactions at high potential regions and the increase in Mn reactions at low potential regions [56]. From Figure 11b–d, it is evident that after 100 cycles, the shifts to lower voltages of the reduction peaks at around 3.8 and 3.5 V diminish with increasing lithium content. This indicates that a higher lithium content helps mitigate capacity fading and voltage decay. This improvement can be attributed to the stabilizing effect of the Li_2_MnO_3_ phase, which contributes to better structural integrity and electrochemical stability during prolonged cycling. The results suggest that optimizing the lithium content in Li-rich cathode materials is a viable strategy to improve their long-term performance.

**Figure 12:**
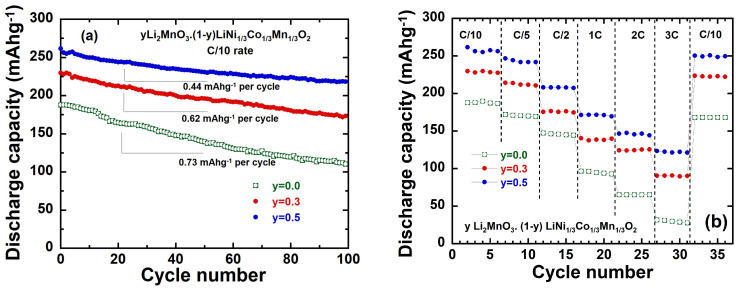
(**a**) Cycling performance at *C*/10 rate and (**b**) rate capability for *y*Li_2_MnO_3_∙(1-*y*) LiNi_1/3_Co_1/3_Mn_1/3_O_2_ electrodes.

The cycling performance of the *y*Li_2_MnO_3_∙(1-*y*)LiNi_1/3_Co_1/3_Mn_1/3_O_2_//Li cells was evaluated at the C/10 rate in the voltage range of 2.0–4.8 V, as shown in Figure 12a. Over extended cycling, a decay in the specific capacity was observed, without significant changes in the characteristic S-shape of the charge and discharge curves (Figure 10d). This capacity decay can be attributed to several factors. First, structural instability arises due to the presence of Mn^3+^ ions, which are prominent in Li-rich cathode materials following the nearly complete activation of the Li_2_MnO_3_ component. Mn^3+^ ions are prone to disproportionation reactions, leading to structural degradation. Additionally, the formation of hydrofluoric acid (HF) in the electrolyte exacerbates the dissolution of Mn, particularly from the spinel-like phase formed during cycling. This dissolution not only reduces the active material but also compromises the cathode’s structural integrity, contributing to the observed capacity fading. From the cycling performance shown in Figure 12a, the capacity loss over 100 cycles is calculated to be 0.73, 0.62, and 0.44 mAh g^−1^ per cycle for electrodes with *y* = 0.0, 0.3, and 0.5, respectively. The retained discharge capacities after 100 cycles in the 2.0–4.8 V potential range follow the following trend:

- Li_1.2_Ni_0.13_Co_0.13_Mn_0.54_O_2,_ with a capacity retention of 83.6%;
- Li_1.134_Ni_0.2_Co_0.2_Mn_0.467_O_2_, with a capacity retention of 75.5%;
- LiNi_1/3_Co_1/3_Mn_1/3_O_2_, with a capacity retention of 60.6%.

Furthermore, the Coulombic efficiency after 100 cycles improves significantly from 60% for the pristine LiNi_1/3_Co_1/3_Mn_1/3_O_2_ with a rhombohedral structure to 84% for Li_1.2_Ni_0.13_Co_0.13_Mn_0.54_O_2_ that includes a 50% Li_2_MnO_3_ cubic structure. These results highlight that an increasing *y*(Li_2_MnO_3_) content enhances the capacity retention and overall cyclability of the cathode materials. The Li_1.2_Ni_0.13_Co_0.13_Mn_0.54_O_2_ electrode, with its high specific capacity and excellent cycling stability, demonstrates superior electrochemical performance compared to other compositions. 

Figure 12b illustrates the rate capability of the *y*Li_2_MnO_3_∙(1-*y*)LiNi_1/3_Co_1/3_Mn_1/3_O_2_ electrodes under various current densities ranging from 0.1C to 3C, in the voltage window 2.0–4.8 V vs. Li^+^/Li. For all three electrode compositions, the discharge capacity is normalized relative to their first discharge capacity at a 0.1C rate, allowing direct comparison. As a general trend, discharge capacity decreases moderately with increasing C rates for all electrodes, demonstrating the impact of higher C rates on the utilization of active materials and lithium-ion diffusion kinetics. Regarding LiNi_1/3_Co_1/3_Mn_1/3_O_2_, this electrode exhibits typical behavior for a pristine *R*-3*m* layered structure. There is a continuous decrease in the discharge capacity as the current rate increases, retaining ~32 mAh g^−1^ when cycled at a high rate of 3C. In contrast to the pristine LiNi_1/3_Co_1/3_Mn_1/3_O_2_, the Li-rich electrodes (*y* = 0.3 and 0.5) demonstrate a significantly better retention of discharge capacity across current rates from 0.1C to 3C. This suggests an activation process that enhances their performance under high-rate conditions. At the 3C rate, the Li_1.134_Ni_0.2_Co_0.2_Mn_0.467_O_2_ and Li_1.2_Ni_0.13_Co_0.13_Mn_0.54_O_2_ electrodes deliver 90 and 123 mAhg^−1^, respectively. The results align well with those reported in the literature [29,34,54,55,56,57]. This excellent rate capability can be attributed to several factors: (i) A well-formed layered structure, as the integrated Li_2_MnO_3_ regions enhance structural stability and facilitate lithium-ion transport during high-rate cycling. (ii) A small particle size: this reduces lithium-ion diffusion paths, promoting faster intercalation/deintercalation reactions [54]. (iii) A high specific surface area: this enhances contact between the active material and the electrolyte, improving reaction kinetics and capacity utilization. Kaewmala et al. [57] highlighted that the increased discharge capacity in the Li_1.2_Ni_0.13_Co_0.13_Mn_0.54_O_2_ electrode is closely associated with its higher *c*/*a* ratio. The elevated *c*/*a* ratio correlates with an increased interslab thickness (*I*_(LiO2)_) as noted in Table 2, facilitating enhanced lithium diffusion. This structural advantage supports larger capacities even at a high current density. Upon reducing the current density back to 0.1C (Figure 12b), the electrode materials retained high discharge capacities: 250.6 mAh g^−1^ (a 95.68% retention of initial capacity) for *y* = 0.5, ~220 mAh g^−1^ (93.34% retention) for *y* = 0.3. For comparison, it is reduced to 168.1mAh g^−1^ (an 89.45% retention) for the pristine material (*y* = 0). These results suggest that the electrode materials, particularly the Li_1.2_Ni_0.13_Co_0.13_Mn_0.54_O_2_ composition, maintain structural integrity and functionality even after extended cycling at high current densities. This resilience is likely due to the robust layered structure and structural benefits provided by the Li_2_MnO_3_ component, ensuring excellent cyclability and capacity retention.

### 3.5. Electrochemical Impedance Spectroscopy (EIS)

The main goal of EIS experiments is a comparison of the electrochemical behavior during long-term cycling and the stability of electrodes comprising “layered–layered” integrated *y*Li_2_MnO_3_∙(1-*y*)LiNi_1/3_C_1/3_Mn_1/3_O_2_ (0.0 ≤ *y* ≤ 0.1) materials. The stability of the electrode materials was studied by comparing the impedance of pristine and Li-rich samples after 100 cycles. Although the three-electrode configuration distinguishes the intrinsic contribution of each individual electrode to the overall battery performance [58], the use of a two-electrode coin Li half-cell can be justified for investigating the electrochemical stability of electrodes. The two-electrode system has some limitations: (i) The potential drop is measured across both the working electrode (WE) and counter electrode (CE), making it difficult to separate the contributions from each electrode and the solution resistance. (ii) The combined impedance of the WE and CE is measured, so it is impossible to distinguish the behavior of individual electrodes. (iii) The measurement includes the uncompensated for solution resistance (iR drop) in the measured impedance, which can obscure the true electrochemical response. In the present work, however, we only utilize EIS to determine the evolution in ohmic and charge transfer resistance and lithium diffusivity among the different cathode materials as a function of their composition, for which the two-electrode system is relevant. Using the same negative electrode configuration, i.e., assuming identical electrode impedance and SEI formation, the main difference in the electrochemical impedance of the Li half-cell comes from the cathode side, for which the charge transfer resistance and the surface film resistance (CEI at the cathode) are the components of the Nyquist plot [16,18,27,34,46,51,53]. 

**Figure 13:**
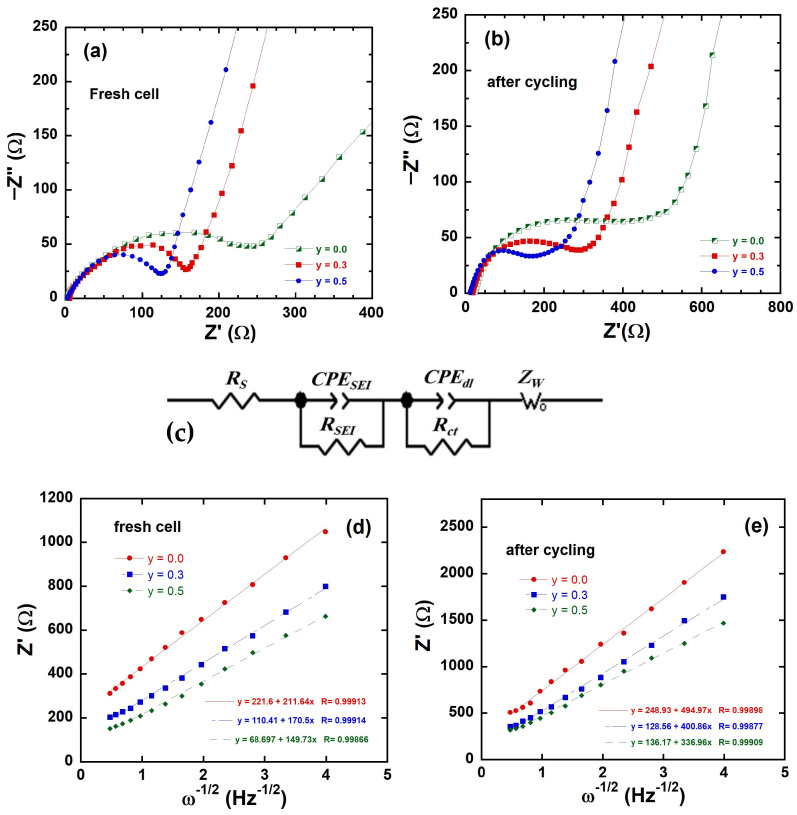
EIS measurements (*Z*″ vs. *Z*′ plots) of *y*Li_2_MnO_3_∙(1-*y*) LiNi_1/3_Co_1/3_Mn_1/3_O_2_ (*y* = 0.0, 0.3, and 0.5) electrodes: (**a**) Fresh electrodes, (**b**) after 50 cycles at a 0.1C rate, (**c**) equivalent model circuit. Plots of the real part of the impedance vs. ω^−1/2^ for (**d**) fresh electrodes and (**e**) after 100 cycles.

Measurements were taken both on fresh cells (before cycling) and after 100 cycles at a 0.1C rate. Nyquist plots of the electrodes, including LiNi_1/3_C_1/3_Mn_1/3_O_2_, Li_1.134_Ni_0.2_Co_0.2_Mn_0.467_O_2_ and Li_1.2_Ni_0.13_Co_0.13_Mn_0.54_O_2_, are presented in Figure 13a–e, with an emphasis on the low-frequency region to analyze diffusion characteristics. The equivalent circuit model used for analyzing the Nyquist plots (as shown in Figure 13c) incorporates four components to represent the processes occurring within the cell: (i) The uncompensated ohmic resistance of the cell (*R*_s_), which is the intercept at high frequency with the *Z*′-axis (horizontal axis). *R_s_* represents the bulk resistance of the cell, including contributions from the electrolyte, current collectors, and cell connections. (ii) The first depressed semicircle in the high-frequency region corresponds to the resistance *R*_SEI_ and capacitance *CPE*_SEI_ associated with the SEI layer formed on the electrode surface. *R*_SEI_ is the resistance to ion transport through the SEI; *CPE*_SEI_ is a constant phase element (CPE) used to model the non-ideal capacitive behavior of the SEI layer. (iii) The second semicircle, appearing in the medium-frequency region, is associated with charge transfer processes at the electrode/electrolyte interface, namely the resistance to charge transfer during the electrochemical reaction (*R*_ct_), and a constant phase element used to describe the non-ideal double-layer capacitance at the interface (*CPE*_dl_). (iv) In the low-frequency region, the inclined line represents the diffusion of lithium ions in the electrode material. This process is characterized by the Warburg impedance Z_W_(ω) = σ_w_ (1 − *j*) ω*^−^*^1/2^, where σ_w_ is the Warburg factor, related to the ion diffusion coefficient; ω is the frequency; and *j* = √−1 [59]. The analysis of the Nyquist plots (Figure 13a,b) reveals the following trends and insights regarding the impedance behavior of the *y*Li_2_MnO_3_∙(1-*y*)LiNi_1/3_C_1/3_Mn_1/3_O_2_ electrodes. (i) A general increase in the total impedance is observed for all electrode materials after the 100th cycle at a 0.1C rate. This increase reflects degradation phenomena, such as growth in the SEI or the accumulation of side reactions with the electrolyte, as expected in the absence of a coating layer known to be essential for Li-rich cathode materials. (ii) The internal ohmic resistances (*R_s_*) are below 10 Ω for fresh cells and remain almost unchanged after cycling. This indicates that the bulk properties of the electrolyte and the current collectors remain stable over the cycling period. (iii) *R*_ct_, associated with the charge transfer process at the electrode/electrolyte interface, is lower for Li-rich materials compared to the parent LiNi_1/3_Co_1/3_Mn_1/3_O_2_ sample. This reduction in *R*_ct_ correlates with the improved electrochemical performance of the Li-rich samples. (iv) The reduced *R*_ct_ can be attributed to the presence of Li_2_MnO_3_ promoting the formation of a spinel phase in the surface layer upon cycling [60]. This formation enhances ionic conductivity and interfacial properties; because the spinel phase possesses 3D channels for Li diffusion, it is expected to have reduced impedance with respect to the layered phase that has only 2D channels [61]. In addition, the incorporation of Li_2_MnO_3_ results in a more stabilized layered structure. This analysis is consistent with the stability of *R*_s_, which suggests that the primary degradation mechanism lies in the interfacial and structural changes of the electrode, rather than bulk electrolyte or contact resistance issues.

The EIS fitting parameters are reported in Table 6. The real part of the impedance *Z*′ (ω) is the sum of the real part of the four components:

*Z*′(ω) = *R*_s_ + *R*_SEI_ + *R*_ct_ + σ_w_ ω^−1/2^ (6)

Figure 13d,e show the plots of the real part of Z vs. ω^−1/2^ of integrated *y*Li_2_MnO_3_∙(1-*y*)LiNi_1/3_C_1/3_Mn_1/3_O_2_ electrodes in the low-frequency range, used to determine the Warburg factor (i.e., the slope of the regression line). The apparent diffusion coefficient *D*_Li_ can be calculated according the following relation [62]:

(7) $$D_{Li}=\frac{R^{2}T^{2}}{2A^{2}n^{4}F^{4}{C_{Li}}^{2}{\sigma_{w}}^{2}},$$

in which *R* is the gas constant, *T* the absolute temperature, *F* the Faraday’s constant, *n* the number of electrons transferred, *C*_Li_ is the concentration of Li^+^ ion inside the electrode, and *A* the effective surface area of the electrode. The values of the apparent diffusion coefficient *D_Li_* before and after cycling are presented in Table 6. It is important to note that for electrodes exhibiting behavior characteristic of multi-phase systems, *D_Li_* is referred to an “apparent” diffusion coefficient. As described by Equation (7), *D_Li_* is predominantly influenced by (1/σ_w_), where a smaller σ_w_ corresponds to a large *D_Li_*. 

As shown in Table 6, the Li-rich electrodes exhibit a lower σ_w_ compared to the pristine electrode LiNi_1/3_C_1/3_Mn_1/3_O_2_, indicating superior ion conductivity and a higher Li^+^ diffusion coefficient. According to data in Table 6, not only the resistances but also the difference in the resistance between the different samples are much larger than the impedance of the Li metal anode. Therefore, the measurement of the potential of the working electrode is not significantly affected by the counter electrode, which eliminates the need for a reference electrode and justifies a posteriori the use of the two-electrode system in the EIS experiments. Indeed, previous experiments already justified the use of the two-electrode configuration. The experimental evidence validating the equivalent circuit in Figure 13c is for instance given in Ref. [63] Li et al. performed EIS measurements on a cathode similar to that of our work with a three-electrode system and demonstrated that the full cell impedance arises predominantly at the positive electrode, that positive electrode data are similar to the full cell data, and that impedance changes at the negative electrode are small [63]. More recently, in their tutorial, Lazanas and Prodrominis [64] explained that when the SEI on the anode plays a role, it generates an inductive loop at a high frequency, which is not observed in our experiments. We can also mention the analysis of Talian et al. [65], reporting that, in lithium batteries, EIS experiments in two-electrode configurations are always technically feasible. Whether the SEI measurements are made with two electrodes [66] or three electrodes [67], which give the same results, the equivalent circuit in our Figure 13c is the same as the one systematically used in the literature for these materials, which is fortunate since it makes possible a comparison between the different modifications and the different synthesis processes that have been used. In particular, this allows us to compare the lithium diffusion coefficient with prior data reported in the literature. The *D_Li_^+^* values fall into the range of 10^−13^ to 10^−12^ cm^2^s^−1^, aligning well with the values reported in the literature, with typically larger Li-layer spacing, reduced Li/Ni cation mixing (as indicated in Table 2), and improved structural stability [27,68,69]. After cycling, there is a slight decrease in *D_Li_*, consistent with increased *R*_ct_ values. This decline can be attributed to electrode aging or the passivation effect of the MnO_2_ species formed during the activation of Li_2_MnO_3_ and their partial incorporation into the electrode surface films [70]. 

The Li-rich electrodes show a decrease in *R_ct_*, which directly indicates an enhanced electron transfer at the electrode/electrolyte interface. The exchange current density (*I*_0_) is calculated using the linearized Butler–Volmer equation [8].

(8) $$I_{0}=\frac{RT}{nF}\frac{1}{R_{ct}}$$

*I*_0_ is an intrinsic property of the cathode material, independent of the cell’s manufacturing process and the size or shape of the particles. The values of the exchange current density *I*_0_ before and after cycling are listed in Table 6. The higher value of *I*_0_ observed in the Li-rich electrodes compared to the pristine materials, even after the 100th cycle, suggests that electrochemical reactions occur more readily on the surface of Li-rich electrodes. Additionally, the apparent diffusion coefficient *D_Li_* of the Li-rich electrodes is greater than that for the pristine electrode, further supporting the conclusion that Li-ion transport and interfacial charge transfer processes are significantly enhanced in the Li-rich materials. This combination of a higher *I*_0_ and improved *D_Li_* underscores the superior electrochemical performance and stability of Li-rich electrodes.

### 3.6. Area-Specific Impedance (ASI)

**Figure 14:**
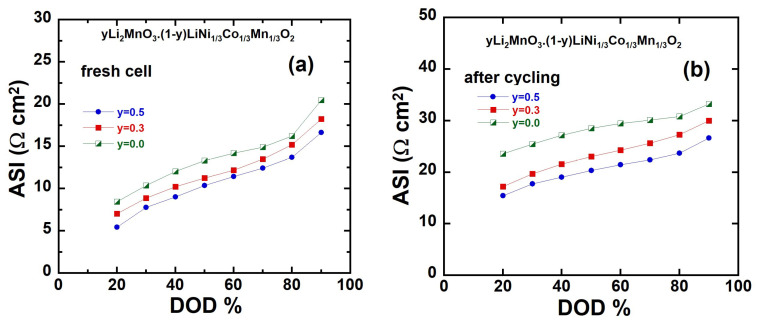
Area-specific impedance (ASI) of parent and *y*Li_2_MnO_3_∙(1-*y*)LiNi_1/3_C_1/3_Mn_1/3_O_2_ (*y* = 0.0, 0.3, and 0.5) as a function of depth of discharge (DOD): (**a**) fresh cell and (**b**) after 100 cycles.

More insights into the variation in the overall cell potential as a function of the depth of charge (DOD) can be gained by evaluating the area-specific impedance (ASI), expressed in Ω cm^2^, which is calculated using the following equation [71]:

(9) $$ASI=A\frac{∆V}{I},$$

where *A* is the cross-sectional area of the electrode, Δ*V* = *OCV*-*V*_cell_ is the potential change during current interruption for 60 s at each DOD, and *I* is the current passed throughout the cell. Various factors can influence the area-specific impedance, including the ohmic drop, Li-ion transport through the electrolyte, and solid-state diffusion within the electrode material. Unlike electrochemical impedance spectroscopy (EIS), ASI does not require equilibrium conditions, making it a more practical and representative technique for evaluating the total internal resistance during cycling. The ASI results corroborate the observations from the EIS measurements. For instance, they highlight the improved ionic and electronic conductivity of Li-rich electrodes, as well as the stabilization of interfacial properties over extended cycling. Additionally, the ASI analysis captures the changes in resistance components due to structural modifications, such as the formation of a spinel-like phase or surface passivation effects. This alignment between ASI and EIS outcomes underscores the reliability of these techniques in assessing the electrochemical performance and stability of battery electrodes. The variation in ASI for the integrated *y*Li_2_MnO_3_∙(1-*y*)LiNi_1/3_C_1/3_Mn_1/3_O_2_ (*y* = 0.0, 0.3, and 0.5) electrodes before and after 100 cycles at a 0.1C rate are illustrated in Figure 14a,b, respectively.

For the fresh cells at 90% DOD (Figure 14a), the measured ASI values are 192, 121, and 77 Ω cm^2^, respectively. After 100 cycles, these values increase to 240, 152, and 106 Ω cm^2^, respectively. These results demonstrate that ASI, and therefore charge transfer resistance, is influenced by both the DOD and the aging of the electrode material. Moreover, after 100 cycles, the pristine electrode (*y* = 0.0) exhibits a much steeper increase in ASI compared to the Li-rich electrodes (*y* = 0.3 and *y* = 0.5). As shown in Figure 14b, the ASI value at 20% DOD for the fresh pristine electrode is about 48 Ω cm^2^, which rises significantly to 133 Ω cm^2^ after 50 cycles. In contrast, the ASI for *y* = 0.3 increases only slightly from 32  to 53 Ω cm^2^, while for *y* = 0.5, the ASI rises modestly from 21  to 32 Ω cm^2^. This suggests that the Li-rich electrodes not only maintain better structural stability but also exhibit superior resistance to aging-related performance degradation. This improved stability can be attributed to the incorporation of Li_2_MnO_3_, which enhances their interfacial properties and promotes the formation of a stabilized layered or spinel-like structure during cycling. However, the Li_1.2_Ni_0.13_Co_0.13_Mn_0.54_O_2_ electrode demonstrates superior performance after cycling. These findings are consistent with our previous studies [29,69] for the Li_1.2_Ni_0.13_Co_0.13_Mn_0.54_O_2_ and Li_1.2_Ni_0.2_Mn_0.6_O_2_electrode, with the results reported by Oh et al. [72] for the Li[Ni_0.5_Mn_0.5_]_1−x_Co_x_O_2_ electrode, and for other Li-rich layer oxides [73].

## 4. Discussion

Li-rich cathode materials have been subject to many investigations. The works prior to 2021 have been reviewed in [74]. Since then, the results reported for Li-rich layered oxide (LLO) cathodes are reported only with modifications involving doping and/or coating, to optimize their electrochemical properties. For example, coating with Li_3_PO_4_ with a spinel structure significantly increases the cycle life by protecting the surface and the rate capability, since Li_3_PO_4_ is conductive [75]. The electrode 0.5Li_2_MnO_3_·0.5LiMn_1/3_Co_1/3_Ni_1/3_O_2_ coated with a Li_3_PO_4_ conductive layer exhibited a capacity of 204.7 mAh g^−1^, with a retention rate of up to 94.4% after 200 cycles at 1C [76]. A LiF-rich cathode–electrolyte interface (CEI) using all-fluorinated electrolyte also improved electrochemical properties [77]. Wang et al. reported how doped Fe^3+^ and Ti^4+^ helped to inhibit the release of lattice oxygen and stabilize the structure of LLOs [78]. Gao et al. synthesized a single crystallized LLO with gradient B doping plus a Li_2_B_4_O_7_ coating [79]. As a cathode, this material exhibited a capacity retention of 87.42% after 300 cycles at 1C, to our knowledge the best performance achived with LLOs. These are only examples of the LLO modification strategies used to address these challenges, elaborated in detail and recently reviewed in [80]. These modifications give evidence of the potential of LLOs as cathode materials for the next generation of Li-ion batteries. We believe that the results obtained on the pristine LLO particles studied in the present work is a promising step and a motivation to apply such modifications to them.

Several researchers have shown that Li-rich layered oxide, 0.5Li_2_MnO_3_∙0.5Li(Ni_1/3_Mn_1/3_Co_1/3_)O_2_, exhibits an interestingly high capacity among several cathode systems. Li et al. [81] reported that the induced rock salt-structure shell significantly restrains lattice oxygen release, TM dissolution, and interfacial side reactions, thereby improving interfacial stability and facilitating Li^+^ diffusion. Combining a powerful synchrotron in situ X-ray diffraction analysis and observations using advanced scanning transmission electron microscopy equipped with a high-angle annular dark-field detector, Ye et al. [82] have revealed that, in Li-rich materials within the Li-Ni-Mn-O system, the sub-reaction of O_2_ generation may feature a much faster kinetics than transition metal diffusion during the Li_2_MnO_3_ activation process, indicating that the latter plays a crucial role in determining the Li_2_MnO_3_ activation rate and leading to an unusual step-wise capacity increase over charging cycles. Li et al. [83] confirmed that the increase in Li_2_MnO_3_ content in a Li-rich cathode does not destroy the high specific capacity brought by nickel ions but achieves a more orderly arrangement of nickel ions between the TM layers and facilitates the diffusion of Li^+^ through the ion channel. This arrangement effectively inhibits the migration of the TM layer and enables the liberated Li^+^ to be re-inserted into the layered crystal lattice, suppressing its irreversible capacity loss and improving its cycle stability.

The superlattice structure in Li_2_MnO_3_ arises from the ordering of lithium and manganese ions in the transition metal layers, with a layered structure (*C*2/*m* space group). It significantly impacts electrochemical properties, because it enhances structural stability during cycling, as it mitigates the large-scale structural distortions caused by lithium intercalation and deintercalation. This effect improves cycle ability. The superlattice structure also facilitates a dual redox mechanism involving both Mn ions (cationic redox) and oxygen (anionic redox) species. This can lead to a higher capacity because the lattice oxygen contributes to reversible redox activity, provided the material is not cycled too aggressively or at too high voltages in order to avoid oxygen release. Note, however, that the superlattice structure in our case is only partial, which is beneficial to lithium mobility, as a complete superlattice structure would restrict pathways for Li^+^ movement, hindering fast diffusion and reducing the rate capability. While the partial superlattice structure in Li_2_MnO_3_ provides unique benefits like a high capacity and structural stability, the partial disorder introduces challenges such as oxygen release and diffusion limitations. The structure of our materials balances these factors and is a key to understand the improvement in their electrochemical performance. In addition, the incorporation of Li_2_MnO_3_ in LiNi_1/3_Co_1/3_Mn_1/3_O_2_ (NCM) has beneficial effects on both the oxygen release and the lithium diffusion. First, it reduces the extent of oxygen release compared to pure Li_2_MnO_3_, as the structural support provided by the NCM phase helps in stabilizing the oxygen sublattice in the Li_2_MnO_3_-derived regions. The NCM component mitigates the large irreversible capacity loss seen in pure Li_2_MnO_3_ during the first charge by providing an additional electrochemically active component that does not undergo oxygen evolution. Second, NCM has better lithium-ion diffusivity due to its less ordered structure compared to the superlattice in Li_2_MnO_3_. Therefore, the combination provides a more balanced structure where the NCM phase acts as a pathway for faster lithium-ion transport. The presence of both phases reduces reliance on the slower lithium diffusion through the superlattice in Li_2_MnO_3_, improving rate capability, while the partial activation of Li_2_MnO_3_ during cycling introduces some disorder, which can further enhance Li^+^ transport. These advantages are thus promising for the future utilization of cathode materials for lithium-ion batteries, even though the rate capability still lags behind more conventional NCM cathodes due to the partial contribution of the slower Li_2_MnO_3_ phase.

## 5. Conclusions

In this work, new stoichiometric, high-voltage, Li-rich integrated cathode materials *y*Li_2_MnO_3_∙(1-*y*)LiNi_1/3_Co_1/3_Mn_1/3_O_2_ (where *y* = 0.0, 0.3, and 0.5) have been synthesized in identical conditions through a sol–gel method assisted by citric acid as a chelating agent, which confirms that Li_2_MnO_3_-rich electrode materials exhibit superior electrochemical performance compared to the conventional LiNi_1/3_Co_1/3_Mn_1/3_O_2_ electrode. The structural and morphological properties of the materials have been characterized using XRD, SEM, HRTEM, EDX, and BET measurements and Raman spectroscopy. The results of this study confirm that Li_2_MnO_3_-rich electrode materials exhibit a superior electrochemical performance compared to the conventional LiNi_1/3_Co_1/3_Mn_1/3_O_2_ electrode. The incorporation of Li_2_MnO_3_ enhances their specific capacity, capacity retention over 100 cycles, and structural stability, while reducing the charge transfer resistance (*R_ct_*) and increasing the apparent lithium-ion diffusion coefficient. The best cathode material 0.5Li_2_MnO_3_∙0.5LiNi_1/3_Co_1/3_Mn_1/3_O_2_ has a capacity retention of 83.6% after 100 cycles in the potential range 2.0–4.8 V vs. Li^+^/Li.

The findings also show that the performance retention after cycling is further improved in lithium-rich electrodes due to the complete activation of the Li_2_MnO_3_ component and the formation of a stabilizing spinel phase at the surface. Moreover, the minimal increase in area-specific impedance (ASI) and *R_ct_* after 100 cycles underscores the enhanced electrochemical durability of these materials. Our results are also due to the good crystalization of the particles, which is known to be an important parameter to achieve good electrochemical performance. Further improvement is expected by coating and doping, following the commonly used process reviewed, for example, in [73]. In addition, although our work gives some insight into SEI formation, further investigations, including XPS, FTIR, and SEM/EDS, are needed for further analysis. In conclusion, Li_2_MnO_3_-rich electrodes represent a promising advancement for lithium-ion batteries, offering higher reaction kinetics, an elevated capacity, and excellent long-term stability, even under high-current conditions. These results pave the way for the targeted optimization of lithium-rich compositions for applied high-energy, long-lasting batteries. 

## Figures and Tables

**Table 1:** Formulation of stoichiometric Li- and Mn-rich layered oxides studied in this work.

*y* Values	Formulation (a)	x Values	Formulation (b)
*y* = 0.0	LiNi_1/3_Co_1/3_Mn_1/3_O_2_	0.33	LiNi_1/3_Co_1/3_Mn_1/3_O_2_
*y* = 0.3	0.3Li_2_MnO_3_∙0.7LiNi_1/3_Co_1/3_Mn_1/3_O_2_	0.20	Li_1.134_Ni_0.2_Co_0.2_Mn_0.466_O_2_
*y* = 0.5	0.5Li_2_MnO_3_∙0.5LiNi_1/3_Co_1/3_Mn_1/3_O_2_	0.13	Li_1.2_Ni_0.13_Co_0.13_Mn_0.54_O_2_

**Table 2:** Structural parameters obtained from Rietveld refinements of X-ray diffractograms of integrated *y*Li_2_MnO_3_∙(1-*y*) LiNi_1/3_Co_1/3_Mn_1/3_O_2_ layered oxides synthesized by citric acid-assisted sol–gel method.

Crystal Data	LiNi_1/3_Co_1/3_Mn_1/3_O_2_	Li_1.134_Ni_0.2_Co_0.2_Mn_0.466_O_2_	Li_1.2_Ni_0.13_Co_0.13_Mn_0.54_O_2_
Lattice parameters			
*a* (Å)	2.858(3)	2.8538 (2)	2.8496 (7)
*c*(Å)	14.217(9)	14.219(3)	14.221(1)
*c*/*a*	4.974(3)	4.982(5)	4.990(9)
*V* (Å^3^)	100.59	100.29	100.01
*R*_1_*= [I_(_*_003)_/*I*_(104)_] ^1^	1.396	1.435	1.520
*R*_2_*= [[I_(_*_006)_ + *I_(_*_012)_ ]/*I*_(101)_] ^a^	0.577	0.473	0.422
L_c_ (nm)	78.3	73.7	63.3
ε × 10^−4^ (rd)	2.7	3.7	5.1
Reliability factors			
*R_p_* (%)	10.26	10.23	9.41
*R_wp_* (%)	11.41	12.11	10.64
*X* ^2^	1.48	1.52	1.46
*Ni*^2+^ % *(in Li layer of R-3m)*	3.10	2.17	1.78
*Z_oxy_*	0.24125	0.24224	0.24648
*S_(MO_*_2*)*_*(Å)* ^b^	2.618	2.591	2.470
*I_(LiO_*_2*)*_*(Å)* ^c^	2.121	2.149	2.270
Phase fraction (mol%)			
*R-3m*	100	70.06	49.18
*C2/m*	0.0	29.94	50.82

^a^ Peak intensity ratios were obtained from normalized patterns. ^b^ *S*_(MO2)_ *=* 2((1/3) − *Z_oxy_*)c is the thickness of the metal–O_2_ planes. ^c^ *I*_(LiO2)_ = *c*/3 − *S*(*MO*_2_) is the thickness of the interslab space.

**Table 3:** Rietveld and EDX analysis of Ni, Co, and Mn in the *y*Li_2_MnO_3_∙(1-*y*) LiNi_1/3_C_1/3_Mn_1/3_O_2_ composite powders in atomic percent (at. %) ratio of elements.

*y*	Theoretical/Experimental		Atomic % Ratio of Elements			Composition as Li[*M*]O_2_ Notation
			Ni	Co	Mn	
0.0	Theoretical		33.33	33.33	33.33	LiNi_1/3_Co_1/3_Mn_1/3_O_2_
	Experimental	Rietveld	33.08	33.71	33.21	LiNi_0.3308_Co_0.3371_Mn_0.3321_O_2_
		EDX	32.89	33.64	33.45	LiNi_0.3289_Co_0.3364_Mn_0.3345_O_2_
0.3	Theoretical		23.00	23.00	53.86	Li_1.134_Ni_0.2_Co_0.2_Mn_0.5386_O_2_
	Experimental	Rietveld	22.44	23.10	54.46	Li_1.134_Ni_0.1957_Co_0.2015_Mn_0.4751_O_2_
		EDX	21.94	23.23	54.80	Li_1.134_Ni_0.1914_Co_0.2029_Mn_0.478_O_2_
0.5	Theoretical		16.25	16.25	67.50	Li_1.2_Ni_0.13_Co_0.13_Mn_0.54_O_2_
	Experimental	Rietveld	16.34	16.51	67.14	Li_1.2_Ni_0.1308_Co_0.1321_Mn_0.5371_O_2_
		EDX	16.52	16.69	66.79	Li_1.2_Ni_0.1322_Co_0.1335_Mn_0.5343_O_2_

**Table 4:** BET results and pore structure parameters for *y*Li_2_MnO_3_∙(1-*y*)LiNi_1/3_C_1/3_Mn_1/3_O_2_ powders.

Composition *y*Li_2_MnO_3_	Specific Surface Area (m^2^ g^−1^)	Average Pore Radius (nm)	Pore Volume (cm^3^ g^−1^)	L_BET_ (nm)	L_SEM_ (nm)
0.0	6.79	3.20	0.0169	185	306
0.3	8.63	3.55	0.0222	157	197
0.5	11.90	3.63	0.0307	119	131

**Table 5:** Theoretical charge, discharge-specific capacities, irreversible capacities (IRs), and Coulombic efficiency (CE) of corresponding components in *y*Li_2_MnO_3_∙(1-*y*)LiNi_1/3_Co_1/3_Mn_1/3_O_2_ based on mass ratio of electrode material compared with observed values corresponding to individual stage.

	Composition		*y* = 0.0	*y* = 0.3	*y* = 0.5
Initial charge (mAh g^−1^)	Theoretical	Total	277.8	332.2	368.3
		*R*-3*m*	277.8	194.5	138.9
		*C*2/*m*	0	137.7	229.4
	Experimental	Total	230.6	307.7	340.8
		*R*-3*m*	230.6	147.2	117.8
		*C*2/*m*	0	160.5	223
Initial discharge (mAh g^−1^)	Theoretical	Total	277.8	280.1	281.6
		*R*-3*m*	277.8	194.5	138.9
		*C*2/*m*	0	85.6	142.7
	Experimental	Total	189	229.4	261.6
		*R*-3*m*	189	147.2	117.8
		*C*2/*m*	0	82.2	152.4
1st cycle	IR (mAh g^−1^)		42.6	78.3	79.2
	CE (%)		81.9	74.5	76.7
2nd cycle	IR (mAh g^−1^)		12	3.5	1.9
	CE (%)		89.3	96.6	98.1
100th cycle	IR (mAh g^−1^)		4.9	2.7	0.1
	CE (%)		95.3	97.7	≈ 100

**Table 6:** Fitting results of Nyquist plots for the *y*Li_2_MnO_3_∙(1-*y*) LiNi_1/3_C_1/3_Mn_1/3_O_2_ (*y*=0.0, 0.3 and 0.5) electrodes before cycling and after 100 cycles.

Parameters	*y* = 0.0		*y* = 0.3		*y* = 0.5	
	Fresh	100th	Fresh	100th	Fresh	100th
*R*_s_ (Ω)	2.01	22.3	4.6	17.7	2.3	12.2
*R*_SEI_ (Ω)	90.8	156.3	53.1	112.6	41.3	56.0
CPE_SEI-T_	4.1 × 10^−5^	1.3 × 10^−4^	3.2 × 10^−5^	1.2 × 10^−5^	2.1 × 10^−6^	4.1 × 10^−6^
CPES_EI-P_	0.73	0.87	0.68	0.82	0.63	0.81
*R_ct_* (Ω)	232.8	454.2	154.1	270.4	121.2	157.5
CPE_dl-T_	4.99 × 10^−5^	2.04 × 10^−4^	3.1 × 10^−5^	7.3 × 10^−5^	2.7 × 10^−5^	4.3 × 10^−5^
CPE_dl-P_	0.91	0.89	0.86	0.67	0.69	0.55
Z_w-R_	210.2	494.1	168.2	399.9	151.6	337.8
Z_w-T_	0.197	31.9	0.161	25.4	0.133	9.5
Z_w-P_	0.303	0.340	0.411	0.394	0.378	0.274
*I*_0_ (µA)	111.3	57.1	148.3	84.5	178.2	128.9
*σ_w_* (Ω s^−1/2^)	211.6	494.9	170.5	400.8	150.7	336.9
*D_Li_*+ (cm^2^ s^−1^)	1.17 × 10^−12^	1.31 × 10^−13^	2.28 × 10^−12^	2.76 × 10^−13^	5.36 × 10^−12^	8.64 × 10^−13^

## Data Availability

Data are contained within the article.
